# Mortality of Philadelphia for October, November and December, 1855; Collated from the Record at the Health Office

**Published:** 1856-02

**Authors:** Wilson Jewell


*Mortality of Philadelphia for October, November and December,* 1855 *; collated from the record at the Health Office. Also, a Summary of deaths for the year.* By WILSON JEWELL, M. D.

The deaths for the 4th quarter of the year, ending December 29th, 1855, embracing a period of 91 days, amount to 2171. Equivalent to an average per diem of $23\frac{4}{5}$ deaths.

By deducting the Still Born, 162, it leaves but 22 deaths per day.

A comparison of the mortality for this quarter, with that for the 3d or last, will present a falling off of 1216, or more than one third.

Hence, in a population, which, at this time may be safely estimated at 500,000, there is the evidence of an unusual state of health.

Nor is it unworthy of notice, that the deaths for this 4th quarter, are less by 28, than for the 4th quarter of 1854.

Compared with the population, we have never presented a more favorable mortality table, since we commenced our statistical labors in 1852.

If we deduct from the total, 162 Still Born children; 88 External Causes; 77 Debility; 37 Old Age, and 43 Unknown—in all 407—there will be left 1764 deaths from legitimate diseases, which will average about 19 deaths per day.

The deaths under one year amount to 422. Under five years 844, or 42 per cent.

More than half of all the deaths or 52.56 per cent. were in children under twenty years.

The deaths from the male sex amount to 1148 ; those from the female sex 1023 ; showing an excess of the former equal to 5·75 per cent.

The deaths over sixty were 228, or 11.34 per cent. of the whole. Of this number, 9 were between 90 and 100, and two were centennarians.

The deaths from Still Born, which we have not included in the above calculations, amounted to 162, or 7.36 per cent. of the total of deaths, and have exceeded those for either of the three preceding quarters.

The deaths from Small Pox have reached 130. One hundred of these, or 77 per cent. occurred in children under ten years of age.

The Zymotic or Epidemic class of diseases, furnish 529 or 26.33 per cent. of the mortality.

The organs of Respiration 481, or 23.94 per cent. The Nervous System 365, or 18.16 per cent. Still Born not included.

The principal diseases from which children under ten years of age have died, were, numerically, as follows :—Convulsions, Small Pox, Croup, Scarlet Fever, Marasmus, Dropsy of the Brain, Debility and Inflammation of Brain.

Sixty-six deaths, averaging 5 per week for the quarter, have been recorded from Scarlet Fever. This disease is on the increase, as is also Small Pox.

With the deaths are included 125 from the Almshouse ; 102 from among the colored population, and 24 from the country. In all 251.

TABLE NO. 1.

*Deaths for the fourth quarter of 1855 classified.*

| | Oct. | Nov. | Dec. | Male. O. | Male. N. | Male. D. | Female. O. | Female. N. | Female. D. | Total. |
|---|---|---|---|---|---|---|---|---|---|---|
| 1 *Endemic & Contagious diseases* Zymotic or Epidemic | 198 | 138 | 193 | 114 | 75 | 103 | 84 | 63 | 90 | 529 |
| 2 *Uncertain or general seat,* Sporadic diseases | 119 | 61 | 77 | 75 | 31 | 39 | 44 | 30 | 38 | 257 |
| 3 Nervous system | 155 | 93 | 117 | 89 | 57 | 72 | 66 | 36 | 45 | 365 |
| 4 Organs of Respiration | 186 | 126 | 169 | 82 | 58 | 77 | 104 | 68 | 92 | 481 |
| 5 " Circulation | 18 | 23 | 18 | 7 | 13 | 10 | 11 | 10 | 8 | 59 |
| 6 Digestive organs | 59 | 37 | 23 | 23 | 20 | 7 | 36 | 17 | 16 | 119 |
| 7 Urinary " | 3 | 3 | 1 | 3 | | 1 | | 3 | | 7 |
| 8 Organs of Generation | 1 | 5 | 9 | | 1 | | 1 | 4 | 9 | 15 |
| 9 " Locomotion | 1 | 3 | 2 | | 1 | 1 | 1 | 2 | 1 | 6 |
| 10 Integumentary system | 2 | 1 | | | | | 2 | 1 | | 3 |
| 11 Old age | 13 | 10 | 14 | 4 | 2 | 3 | 9 | 8 | 11 | 37 |
| 12 External causes | 33 | 25 | 30 | 22 | 17 | 23 | 11 | 8 | 7 | 88 |
| Still Born | 59 | 56 | 47 | 37 | 31 | 24 | 22 | 25 | 23 | 162 |
| Unknown | 20 | 6 | 17 | 11 | 5 | 10 | 9 | 1 | 7 | 43 |
| | 867 | 587 | 717 | 467 | 311 | 370 | 400 | 276 | 347 | 2171 |

TABLE NO. 2.

1. *Endemic and Contagious Diseases—Zymotic or Epidemic.*

| | Male. | Female. | Under 1 yr. | 1 to 2. | 2 to 5. | 5 to 10. | 10 to 15. | 15 to 20. | 20 to 30. | 30 to 40. | 40 to 50. | 50 to 60. | 60 to 70. | 70 to 80. | 80 to 90. | 90 to 100. | 100 to 110. | Total. |
|---|---|---|---|---|---|---|---|---|---|---|---|---|---|---|---|---|---|---|
| Cholera | 3 | 1 | | | 1 | | | | | 2 | 1 | | | | | | | 4 |
| " infantum | 9 | 6 | 12 | 2 | 1 | | | | | | | | | | | | | 15 |
| Croup | 44 | 43 | 9 | 13 | 47 | 16 | 2 | | | | | | | | | | | 87 |
| Diarrhœa | 10 | 16 | 6 | 2 | 1 | 1 | | | 1 | 3 | 2 | 5 | 3 | 1 | 1 | | | 26 |
| Dysentery | 18 | 9 | 3 | 2 | 1 | 1 | | | 4 | 4 | 4 | 3 | 4 | 1 | | | | 27 |
| Erysipelas | 8 | 1 | 2 | | | 1 | | | | 2 | 2 | | 1 | 1 | | | | 9 |
| Fever, | 2 | | | | 1 | | | | | | 1 | | | | | | | 2 |
| " Bilious | 7 | 4 | | | 1 | 1 | | 1 | 1 | 3 | 1 | 1 | 1 | 1 | | | | 11 |
| " Congestive | 2 | 1 | | | | | | 1 | 1 | | 1 | | | | | | | 3 |
| " Gastric | 3 | 1 | 1 | | 1 | 1 | | | | 1 | | | | | | | | 4 |
| " Hectic | 1 | 1 | 1 | 1 | | | | | | | | | | | | | | 2 |
| " Intermittent | 1 | 1 | | 1 | 1 | | | | | | | | | | | | | 2 |
| " Malignant | 1 | | | | | | | | 1 | | | | | | | | | 1 |
| " Nervous | | 1 | | | | | | | 1 | | | | | | | | | 1 |
| " Remittent | 14 | 5 | | | 1 | | 2 | 3 | 6 | 5 | 1 | 1 | | | | | | 19 |
| " Scarlet | 31 | 35 | 2 | 19 | 29 | 14 | 2 | | | | | | | | | | | 66 |
| " Typhoid | 39 | 39 | 1 | | 3 | 6 | 2 | 10 | 25 | 14 | 4 | 7 | 4 | 2 | | | | 78 |
| " Typhus | 5 | 5 | | | | | | | 4 | 3 | 2 | 1 | | | | | | 10 |
| " Yellow | 1 | | | | | | | | 1 | | | | | | | | | 1 |
| Hooping Cough | 10 | 8 | 12 | 3 | 2 | 1 | | | | | | | | | | | | 18 |
| Measles | 3 | 1 | | | 3 | 1 | | | | | | | | | | | | 4 |
| Small Pox | 75 | 55 | 26 | 18 | 42 | 14 | 3 | 3 | 9 | 7 | 5 | 2 | 1 | | | | | 130 |
| Syphilis | | 1 | 1 | | | | | | | | | | | | | | | 1 |
| Varioloid | 5 | 3 | 3 | 1 | 4 | | | | | | | | | | | | | 8 |
| | 292 | 237 | 79 | 62 | 139 | 57 | 11 | 18 | 54 | 44 | 24 | 20 | 14 | 6 | 1 | | | 529 |

### 2. *Uncertain or General Seat—Sporadic Diseases.*

| | Male. | Female. | Under 1 yr. | 1 to 2. | 2 to 5. | 5 to 10. | 10 to 15. | 15 to 20. | 20 to 30. | 30 to 40. | 40 to 50. | 50 to 60. | 60 to 70. | 70 to 80. | 80 to 90. | 90 to 100. | 100 to 110. | Total. |
|---|---|---|---|---|---|---|---|---|---|---|---|---|---|---|---|---|---|---|
| Abscess | 5 | 3 | | 1 | 1 | | | | 1 | 1 | | 2 | 1 | 1 | | | | 8 |
| Cancer | 1 | 3 | | | | | | | | 1 | | 1 | 1 | 1 | | | | 4 |
| " Breast | | 1 | | | | | | | | | | 1 | | | | | | 1 |
| Cyanosis | 7 | 2 | 8 | 1 | | | | | | | | | | | | | | 9 |
| Debility | 41 | 36 | 33 | 3 | 3 | 2 | | | 1 | 3 | 5 | 5 | 10 | 7 | 4 | 1 | | 77 |
| Dropsy | 14 | 12 | | 2 | 3 | 2 | | 1 | 3 | 4 | 3 | 3 | 3 | 2 | | | | 26 |
| Fungus Hematodes | | 2 | | | | | | | | | | 1 | 1 | | | | | 2 |
| Gangrene | | 2 | 1 | | | | | | | 1 | | | | | | | | 2 |
| Gout | 2 | | | | | | | | | 1 | | | | 1 | | | | 2 |
| Hemorrhage | 1 | | | | | | | | | | 1 | | | | | | | 1 |
| Inanition | 9 | 12 | 19 | | | 1 | | | | | | | 1 | | | | | 21 |
| Inflammation, Mouth | 1 | | | | 1 | | | | | | | | | | | | | 1 |
| " Throat | 1 | 2 | 1 | 1 | 1 | | | | | | | | | | | | | 3 |
| Malformation | 44 | | 4 | | | | | | | | | | | | | | | 4 |
| Marasmus | 6 | 27 | 42 | 8 | 9 | 1 | | | 1 | 1 | 2 | 3 | 2 | 2 | 1 | | 1 | 73 |
| Mortification | 1 | 1 | | | | | | | | | | | 1 | 1 | | | | 2 |
| Scirrhus | 1 | | | | | | | | | | | | 1 | | | | | 1 |
| Scrofula | 7 | 7 | 3 | 1 | 1 | 3 | 1 | 1 | 1 | 2 | 1 | | | | | | | 14 |
| Sore Throat | 1 | | | | 1 | | | | | | | | | | | | | 1 |
| Ulceration, Tonsils | 1 | | | | | | | | | 1 | | | | | | | | 1 |
| " Throat | 2 | 2 | 1 | 2 | 1 | | | | | | | | | | | | | 4 |
| | 145 | 112 | 112 | 19 | 21 | 9 | 1 | 2 | 7 | 15 | 12 | 16 | 21 | 15 | 5 | 1 | 1 | 257 |

### 3. *Nervous Diseases.*

| | Male. | Female. | Under 1 yr. | 1 to 2. | 2 to 5. | 5 to 10. | 10 to 15. | 15 to 20. | 20 to 30. | 30 to 40. | 40 to 50. | 50 to 60. | 60 to 70. | 70 to 80. | 80 to 90. | 90 to 100. | 100 to 110. | Total. |
|---|---|---|---|---|---|---|---|---|---|---|---|---|---|---|---|---|---|---|
| Apoplexy | 18 | 7 | | | | | | 1 | 2 | 3 | 3 | 3 | 9 | 3 | | 1 | | 25 |
| Concussion of Brain | 1 | | | | | | | | | | | 1 | | | | | | 1 |
| Congestion " | 20 | 20 | 10 | 4 | 7 | 3 | | 3 | 4 | 2 | 3 | 1 | 3 | | | | | 40 |
| Coma | 2 | | | | | | | | | 1 | | 1 | | | | | | 2 |
| Convulsions | 79 | 53 | 73 | 23 | 22 | 6 | 1 | 2 | | 3 | 1 | 1 | | | | | | 132 |
| Disease of Brain | 8 | 8 | 7 | 5 | 1 | | | | | 1 | | 2 | | | | | | 16 |
| Dropsy " | 29 | 17 | 19 | 12 | 7 | 4 | 1 | 1 | | | | 1 | | 1 | | | | 46 |
| Effusion " | 7 | 5 | 4 | 1 | 4 | 1 | | | 2 | | | | | | | | | 12 |
| Epilepsy | 2 | 3 | 2 | | | | | | 1 | | 1 | 1 | | | | | | 5 |
| Inflammation of Brain | 28 | 23 | 17 | 7 | 7 | 4 | 2 | 1 | 3 | 2 | 5 | 2 | 1 | | | | | 51 |
| " Tympanum | 1 | | 1 | | | | | | | | | | | | | | | 1 |
| Mania | 1 | | | | | | | | | | 1 | | | | | | | 1 |
| " a Potu | 4 | 1 | | | | | | | | | 2 | 3 | | | | | | 5 |
| Neuralgia | 1 | | | | | | | | | | | | | 1 | | | | 1 |
| Palsy | 10 | 8 | | | | | 1 | 1 | 2 | 1 | 2 | 5 | 1 | 3 | 2 | | | 18 |
| Softening of Brain | 3 | | | 1 | | | | | 1 | | | 1 | | | | | | 3 |
| Tetanus | 4 | | | | 1 | | 1 | | 1 | | 1 | | | | | | | 4 |
| Trismus | | 2 | 2 | | | | | | | | | | | | | | | 2 |
| | 218 | 147 | 135 | 53 | 49 | 18 | 6 | 9 | 16 | 13 | 19 | 22 | 14 | 8 | 2 | 1 | | 365 |

4. *Organs of Respiration.*

| | Male. | Female. | Under 1 yr. | 1 to 2. | 2 to 5. | 5 to 10. | 10 to 15. | 15 to 20. | 20 to 30. | 30 to 40. | 40 to 50. | 50 to 60. | 60 to 70. | 70 to 80. | 80 to 90. | 90 to 100. | 100 to 110. | Total. |
|---|---|---|---|---|---|---|---|---|---|---|---|---|---|---|---|---|---|---|
| Abscess of Lungs | 1 | | | | | | | | 1 | | | | | | | | | 1 |
| Asthma | 2 | 3 | | | | | | | | 1 | 3 | | | 1 | | | | 5 |
| Congestion of Lungs | 5 | 9 | 1 | 2 | 1 | 1 | | | 1 | 2 | | 3 | 1 | 2 | | | | 14 |
| Consumption " | 123 | 187 | 5 | 1 | 6 | 5 | 3 | 40 | 87 | 74 | 38 | 26 | 19 | 4 | 2 | | | 310 |
| Disease of " | 3 | 3 | | | | | | 2 | | 1 | 1 | 1 | 1 | | | | | 6 |
| " Chest | 1 | 1 | | | 1 | | | | | | | | | 1 | | | | 2 |
| Dropsy of " | 2 | 6 | | 1 | 1 | | | 1 | 1 | 2 | | | 1 | 1 | | | | 8 |
| " Lungs | 1 | | | | | | | | | 1 | | | | | | | | 1 |
| Effusion of Chest | 1 | 1 | | | | | | | 1 | | 1 | | | | | | | 2 |
| Hemorrhage of Lungs | 5 | 2 | | | | | | | 3 | 2 | | 1 | | 1 | | | | 7 |
| Inflamm'n of Bronchi | 27 | 18 | 19 | 5 | 6 | 3 | | 1 | | 2 | 3 | 2 | 3 | | 1 | | | 45 |
| " Chest | 1 | 2 | 1 | | 1 | | | | | | 1 | | | | | | | 3 |
| " Larynx | 1 | 1 | 2 | | | | | | | | | | | | | | | 2 |
| " Lungs | 41 | 30 | 18 | 9 | 14 | 3 | | 1 | 4 | 4 | 5 | 5 | 2 | 4 | 1 | 1 | | 71 |
| " Pleura | 3 | 1 | | | | 1 | | | | 1 | 1 | | | 1 | | | | 4 |
| | 217 | 264 | 46 | 18 | 30 | 13 | 3 | 45 | 98 | 90 | 53 | 38 | 27 | 15 | 4 | 1 | | 481 |

5. *Organs of Circulation.*

| | Male. | Female. | Under 1 yr. | 1 to 2. | 2 to 5. | 5 to 10. | 10 to 15. | 15 to 20. | 20 to 30. | 30 to 40. | 40 to 50. | 50 to 60. | 60 to 70. | 70 to 80. | 80 to 90. | 90 to 100. | 100 to 110. | Total. |
|---|---|---|---|---|---|---|---|---|---|---|---|---|---|---|---|---|---|---|
| Anæmia | 2 | 1 | | | | | | | 1 | 2 | | | | | | | | 3 |
| Aneurism | 1 | 1 | | | | | | | | 1 | | 1 | | | | | | 2 |
| Aneurism of Aorta | 1 | | | | | | | | 1 | | | | | | | | | 1 |
| Angina Pectoris | 1 | | | | | | | | | | | | 1 | | | | | 1 |
| Disease of Heart | 17 | 17 | 4 | | 2 | 1 | | 2 | 1 | 8 | 1 | 6 | 5 | 4 | | | | 34 |
| Dropsy " | 2 | | | | | | | | | | 2 | | | | | | | 2 |
| Enlargem't " | 3 | 7 | | 1 | | | | | 4 | 1 | | 1 | 2 | | 1 | | | 10 |
| Inflammat'n " | 3 | 1 | | | | | | | 1 | 1 | 1 | | | | 1 | | | 4 |
| Leucocythemia | | 1 | | | | | | | 1 | | | | | | | | | 1 |
| Ossification of Arteries | | 1 | | | | | | | | | | | | 1 | | | | 1 |
| | 30 | 29 | 4 | 1 | 2 | 1 | | 2 | 9 | 13 | 4 | 8 | 8 | 5 | 2 | | | 59 |

6. *Digestive Organs.*

| | Male. | Female. | Under 1 yr. | 1 to 2. | 2 to 5. | 5 to 10. | 10 to 15. | 15 to 20. | 20 to 30. | 30 to 40. | 40 to 50. | 50 to 60. | 60 to 70. | 70 to 80. | 80 to 90. | 90 to 100. | 100 to 110. | Total. |
|---|---|---|---|---|---|---|---|---|---|---|---|---|---|---|---|---|---|---|
| Abscess of Liver | 1 | | | | | | | | | 1 | | | | | | | | 1 |
| Cancer of Stomach | 3 | | | | | | | | | 1 | | 1 | 1 | | | | | 3 |
| Cancrum Oris | | 1 | | | 1 | | | | | | | | | | | | | 1 |
| Cirrhosis of Liver | 1 | | | | | | | | | 1 | | | | | | | | 1 |
| Colic | 1 | 1 | | | | | | | | | 1 | | 1 | | | | | 2 |
| Congestion of Bowels | | 1 | 1 | | | | | | | | | | | | | | | 1 |
| " Liver | | 1 | | | | | | | | | | | 1 | | | | | 1 |
| Constipation | | 2 | 2 | | | | | | | | | | | | | | | 2 |
| Cramp | 3 | | 1 | | 1 | | | | 1 | | | | | | | | | 3 |
| Disease of Bowels | 1 | | 1 | | | | | | | | | | | | | | | 1 |
| " Liver | | 2 | | | 1 | | | | | 1 | | | | | | | | 2 |
| " Stomach | 2 | 3 | | | | 1 | | | | 1 | 1 | 1 | 1 | | | | | 5 |
| Dropsy ,Abdominal | 1 | 7 | | | | | | | | 1 | 1 | 2 | 1 | 3 | | | | 8 |
| Enlargement of Liver | 1 | 1 | | | | | | | | 1 | | 1 | | | | | | 2 |
| Hemorrhage of Bowels | 2 | | | | 1 | | | | | | 1 | | | | | | | 2 |
| Hemorrhoids | | 1 | | | | | | | | 1 | | | | | | | | 1 |
| Hernia | 1 | | | | | | | | | | | 1 | | | | | | 1 |
| Ileus | | 1 | | | | | | | | | | | 1 | | | | | 1 |
| Inflammation of Abdomen | | 1 | 1 | | | | | | | | | | | | | | | 1 |
| " Bowels | 9 | 7 | 2 | 3 | 1 | 1 | 2 | | 1 | 2 | 1 | | 2 | 1 | | | | 16 |
| " Liver | | 5 | 1 | | | | | | 1 | 2 | | | 1 | | | | | 5 |
| " Peritoneum | 3 | 8 | | | 1 | 1 | | | 4 | 3 | 1 | | 1 | | | | | 11 |
| " Stomach | 10 | 8 | 1 | 1 | 2 | | 1 | | | 2 | 1 | 1 | 5 | 2 | 2 | | | 18 |
| " Stom. & Bowels | 1 | | | | | 1 | | | | | | | | | | | | 1 |
| Jaundice | 1 | 3 | | | | | | | 1 | 3 | | | | | | | | 4 |
| Mortification of Bowels | | 1 | | 1 | | | | | | | | | | | | | | 1 |
| Obstruction " | 2 | 3 | 1 | | | 1 | | | | 1 | | 1 | | 1 | | | | 5 |
| Scirrhus Stomach | 2 | | | | | | | | | | 1 | | | 1 | | | | 2 |
| Stomatitis | | 1 | 1 | | | | | | | | | | | | | | | 1 |
| Tabes Mesenterica | 1 | 3 | 2 | 2 | | | | | | | | | | | | | | 4 |
| Teething | | 4 | 2 | 2 | | | | | | | 1 | | | | | | | 4 |
| Tumor in Stomach | | 1 | | | | | | | | | 1 | | | | | | | 1 |
| Ulceration of Bowels | 1 | 3 | | 1 | | | 1 | | 1 | | | | | | | | | 4 |
| " Stomach | 2 | | | | | | | | | | | | 1 | | | | | 2 |
| Worms | 1 | | 1 | | | | | | 1 | | | | | | | | | 1 |
| | 50 | 69 | 17 | 10 | 8 | 5 | 4 | | 10 | 21 | 10 | 8 | 16 | 8 | 2 | | | 119 |

7. *Urinary Organs.*

| | Male. | Female. | Under 1 yr. | 1 to 2. | 2 to 5. | 5 to 10. | 10 to 15. | 15 to 20. | 20 to 30. | 30 to 40. | 40 to 50. | 50 to 60. | 60 to 70. | 70 to 80. | 80 to 90. | 90 to 100. | 100 to 110. | Total. |
|---|---|---|---|---|---|---|---|---|---|---|---|---|---|---|---|---|---|---|
| Albuminuria | | 1 | | | | | | | | 1 | | | | | | | | 1 |
| Diabetes | | 1 | | | | | | | | | | 1 | | | | | | 1 |
| Inflammation of Bladder | 1 | | | | | | | | | | | 1 | | | | | | 1 |
| " Kidneys | 1 | | | | 1 | | | | | | | | | | | | | 1 |
| Ischuria Renalis | 1 | | | | | | | | | | | 1 | | | | | | 1 |
| Suppression Urine | | 1 | | | | | | | | | | 1 | | | | | | 1 |
| Rupture Urethra | 1 | | | | | | | | 1 | | | | | | | | | 1 |
| | 4 | 3 | | | 1 | | | | 1 | 1 | | 4 | | | | | | 7 |

8. *Organs of Generation.*

| | Male. | Female. | Under 1 yr. | 1 to 2. | 2 to 5. | 5 to 10. | 10 to 15. | 15 to 20. | 20 to 30. | 30 to 40. | 40 to 50. | 50 to 60. | 60 to 70. | 70 to 80. | 80 to 90. | 90 to 100. | 100 to 110. | Total. |
|---|---|---|---|---|---|---|---|---|---|---|---|---|---|---|---|---|---|---|
| Chlorosis | | 1 | | | | | | 1 | | | | | | | | | | 1 |
| Disease of Uterus | | 1 | | | | | | | | | | | | 1 | | | | 1 |
| Hemorrhage " | | 1 | | | | | | | 1 | | | | | | | | | 1 |
| Inflammation " | | 1 | | | | | | | 1 | | | | | | | | | 1 |
| " Prostrate Gland | 1 | | | | | | | | | | | | | 1 | | | | 1 |
| Laceration of Uterus | | 1 | | | | | | | 1 | | | | | | | | | 1 |
| Ovarian Tumor | | 1 | | | | | | | | | 1 | | | | | | | 1 |
| Puerperal Convulsions | | 2 | | | | | | | 1 | | | 1 | | | | | | 2 |
| " Fever | | 6 | | | | | | | 5 | 1 | | | | | | | | 6 |
| | 1 | 14 | | | | | | 1 | 9 | 1 | 1 | 1 | | 2 | | | | 15 |

9. *Organs of Locomotion.*

| | Male. | Female. | Under 1 yr. | 1 to 2. | 2 to 5. | 5 to 10. | 10 to 15. | 15 to 20. | 20 to 30. | 30 to 40. | 40 to 50. | 50 to 60. | 60 to 70. | 70 to 80. | 80 to 90. | 90 to 100. | 100 to 110. | Total. |
|---|---|---|---|---|---|---|---|---|---|---|---|---|---|---|---|---|---|---|
| Caries | | 1 | | | | | | | | 1 | | | | | | | | 1 |
| " of Spine | | 1 | | | 1 | | | | | | | | | | | | | 1 |
| Disease of Hip | 1 | | | | | | | | 1 | | | | | | | | | 1 |
| " Spine | 1 | 2 | 1 | | | | 1 | | 1 | | | | | | | | | 3 |
| | 2 | 4 | 1 | | 1 | | 1 | | 2 | 1 | | | | | | | | 6 |

10. *Integumentary System.*

| | Male. | Female. | Under 1 yr. | 1 to 2. | 2 to 5. | 5 to 10. | 10 to 15. | 15 to 20. | 20 to 30. | 30 to 40. | 40 to 50. | 50 to 60. | 60 to 70. | 70 to 80. | 80 to 90. | 90 to 100. | 100 to 110. | Total. |
|---|---|---|---|---|---|---|---|---|---|---|---|---|---|---|---|---|---|---|
| Herpes | | 1 | | | | | | | | 1 | | | | | | | | 1 |
| Purpura Hemorrhagica | | 2 | 1 | | 1 | | | | | | | | | | | | | 2 |
| | | 3 | 1 | | 1 | | | | | 1 | | | | | | | | 3 |

11. *Old Age.*

| | | | | | | | | | | | | | | | | | | |
|---|---|---|---|---|---|---|---|---|---|---|---|---|---|---|---|---|---|---|
| Old Age | 9 | 28 | | | | | | | | | | | | 11 | 19 | 6 | 1 | 37 |

12. *External Causes.*

| | Male. | Female. | Under 1 yr. | 1 to 2. | 2 to 5. | 5 to 10. | 10 to 15. | 15 to 20. | 20 to 30. | 30 to 40. | 40 to 50. | 50 to 60. | 60 to 70. | 70 to 80. | 80 to 90. | 90 to 100. | 100 to 110. | Total. |
|---|---|---|---|---|---|---|---|---|---|---|---|---|---|---|---|---|---|---|
| Asphyxia | 1 | 6 | 7 | | | | | | | | | | | | | | | 7 |
| Burns | 3 | 2 | | 2 | 2 | | | | 1 | | | | | | | | | 5 |
| Casualties | 28 | 3 | 3 | | | | 1 | | 7 | 9 | 6 | 4 | 1 | | | | | 31 |
| Drowned | 11 | 4 | 2 | 1 | | 1 | 1 | 1 | | 5 | 4 | | | | | | | 15 |
| Exposure | 2 | 1 | 1 | | | | | | | 1 | | | 1 | | | | | 3 |
| Fracture | 2 | | 1 | | | | | | | 1 | | | | | | | | 2 |
| Intemperance | 6 | 6 | | | | | | | 2 | 2 | 3 | 4 | 1 | | | | | 12 |
| Poisoning | | 1 | | | | 1 | | | | | | | | | | | | 1 |
| Suffocation | | 2 | | | | 1 | | | 1 | | | | | | | | | 2 |
| Suicide | 7 | 1 | | | | | | | 1 | 2 | 4 | 1 | | | | | | 8 |
| Violence | 1 | | | | | | | | | 1 | | | | | | | | 1 |
| Wound | 1 | | | | | | | | | | | 1 | | | | | | 1 |
| | 62 | 26 | 14 | 3 | 2 | 3 | 2 | 1 | 12 | 21 | 17 | 10 | 3 | | | | | 88 |
| Still Born | 92 | 70 | 162 | | | | | | | | | | | | | | | 162 |
| Unknown | 26 | 17 | 13 | | 2 | | | | 4 | 7 | 6 | 4 | 5 | 2 | | | | 43 |

TABLE NO. 3.

*Deaths for the Fourth Quarter of* 1855, *at fifteen distinct periods of life*

| | |
|---|---|
| Under 1 year, | 422 |
| 1 to 2 | 166 |
| 2 to 5 | 256 |
| 5 to 10 | 106 |
| 10 to 15 | 28 |
| 15 to 20 | 78 |
| 20 to 30 | 222 |
| 30 to 40 | 228 |
| 40 to 50 | 146 |
| 50 to 60 | 129 |
| 60 to 70 | 109 |
| 70 to 80 | 72 |
| 80 to 90 | 36 |
| 90 to 100 | 9 |
| 100 to 110 | 2 |
| | 2009 |
| Still Born | 162 |
| Total, | 2171 |

Included within the above table are 125 from the Blockley Alms House; 102 Blacks, and 24 from the country as follows:

| | Oct. | Nov. | Dec. | Total. |
|---|---|---|---|---|
| Almshouse, | 40 | 37 | 48 | 125 |
| Blacks, | 50 | 26 | 26 | 102 |
| Country, | 16 | 5 | 3 | 24 |
| | 106 | 68 | 77 | 251 |

### *General Summary of Deaths for* 1855.

The mortality for the city during the year, in accordance with the returns made to the Health office, has been 10,458; less by 1326, or 5·96 per cent. than the mortality for 1854. This reduced total, may to some extent, be accounted for, from the absence of any epidemic during the year.

According to the estimated population of our city, which embraces in its consolidated organization, the entire county of Philadelphia, and which we have set down at 500,000,* the aggregate of 10,458, will give us one death in every 47·81 of the population, or 20·91 in each thousand. The average monthly mortality during the year, was 871½; weekly 201, and daily 28⅝.

The highest mortality occurred in August, amounting to 1,455 deaths, averaging 47 per day. The lowest was in November, 587 or 19 per day.

Out of the whole number of deaths 3,245, or 31 per cent. took place within the 1st year of life. Before the fifth year 5280, or 50·48 per cent., and previous to the expiration of the twentieth year, 6146, or 58·76 per cent.

Ten hundred and sixty-six, or 10·19 per cent. of the deaths occurred within the third decade of life, or between 20 and 30 years. One hundred and ninety-one, or 1·82 per cent. were among the octogenarians. Forty-nine were nonangenarians. Six were centennarians, and of these, one had lived beyond 110 years. The above calculations include the Still-born.

By deducting the number that have died from causes unknown, from External causes, Old age, Malformations and Debility, as also the Still-Born and those from the country, which in the aggregate, amount to 1859, from the total of deaths for the year, there will be left only 8465 deaths from legitimately accredited diseases, which when averaged, would give but 23·46 deaths for each day, or 16·93 for every thousand of the population.

The following table will afford at a single glance, the yearly mortality in a classified form, together with the rate of percentage compared with the whole number of deaths.

* By some statisticians, the population of our city would be estimated at 525,000. We prefer, however, to take the less number, as the safest calculation, basing it upon an increase of four per cent. per annum, since the census of 1850, when the population of the county was fixed at 408,762.

| | Yearly Mortality. | Per cent. to whole amount. |
|---|---|---|
| 1. Endemic and Contagious diseases, Zymotic or Epidemic, . . . . | 2460 | 23·52 |
| 2. Uncertain or General seat, Sporadic diseases, . . . . . | 1374 | 13·13 |
| 3. Nervous System, . . . . . | 1883 | 18· |
| 4. Organs of Respiration, . . . | 2295 | 21·94 |
| 5. " Circulation, . . . . | 265 | 2·53 |
| 6. " Digestion, . . . . | 598 | 5·70 |
| 7. Urinary Organs, . . . . | 34 | 0·32 |
| 8. Organs of Generation, . . . | 76 | 0 72 |
| 9. " Locomotion, . . . . | 30 | 0·28 |
| 10. Integumentary System, . . . | 20 | 0·19 |
| 11. Old Age, . . . . . . | 173 | 1·65 |
| 12. External causes, . . . . | 442 | 4·22 |
| Still-Born, . . . . . . | 588 | 5·62 |
| Unknown. . . . . . . | 220 | 2·10 |

According to this classified arrangement, the Endemic or Epidemic diseases exhibit, as usual, the highest mortality, viz: 2460, equivalent to 23·52 per cent. of the whole number of deaths for the year. The next highest rate are those of the organs of Respiration, 2295, or 21.94 per cent. of the whole. The diseases of the Nervous System are next in order, amounting to 1883, or 18 per cent. Then follows the Sporadic class, numbering 1374, or 13·13 per cent. Old Age furnishes 1·65 per cent. of the mortality. The deaths from External causes, make 4·22 per cent. Still-Born adds 5·62 per cent., while those deaths set down as Unknown, constitute 2·10 per cent. of the annual mortuary record.

Among the diseases of the organs of Respiration, we find Pulmonary Consumption largely represented, numbering 1327. Thus contributing more than half, or 64·44 per cent. of all the deaths charged to this class. Of the aggregate of deaths from all causes for the year, exclusive of Still-Born, it constituted more than 13·44 per cent. 1 per cent. more than for 1854. While for every thousand of the population it carried off 2·65.

As in former statistics, so in these, this disease not only furnishes a large outlet to life, but invades every age, from infancy to those of three score years and ten, not exempting either sex. Thirty-six were under one year of age. Those under the age of twenty amounted to 239. The highest mortality in any decade of life, was between 20 and 30, viz: 427, or 32·17 per cent. Forty were over 70 years of age. The excess of deaths in the sexes, was on the side of females, equal to 5·65 per cent.

The following is a synopsis of those diseases which have been most fatal, and, therefore, supposed to be most prevalent, for eight years in this city :—

| | 1848. | 1849. | 1850. | 1851. | 1852. | 1853. | 1854. | 1855. |
|---|---|---|---|---|---|---|---|---|
| Cholera Infantum - - - - - - - | 454 | 582 | 505 | 397 | 329 | 398 | 715 | 566 |
| Consumption of Lungs - - - - | 965 | 939 | 907 | 881 | 1204 | 1246 | 1394 | 1327 |
| Congestion of Brain - - - - - | 85 | 91 | 97 | 130 | 120 | 172 | 222 | 175 |
| Convulsions - - - - - - - - - | 401 | 415 | 444 | 479 | 499 | 543 | 695 | 626 |
| Croup - - - - - - - - - - - - | 177 | 130 | 143 | 180 | 208 | 303 | 304 | 265 |
| Diarrhœa - - - - - - - - - - | 122 | 225 | 208 | 157 | 156 | 130 | 219 | 177 |
| Dropsy of the Brain - - . - - | 220 | 237 | 283 | 245 | 247 | 192 | 299 | 254 |
| Dysentery - - - - - - - - - | 315 | 578 | 421 | 401 | 558 | 379 | 446 | 266 |
| Inflammation of the Brain - - - | 186 | 198 | 218 | 202 | 258 | 157 | 362 | 288 |
| " " Bronchi - - | 172 | 169 | 191 | 175 | 208 | 197 | 243 | 233 |
| " " Lungs - - - | 265 | 273 | 352 | 352 | 444 | 353 | 472 | 442 |
| Marasmus - - - - - - - - - | 237 | 264 | 217 | 255 | 354 | 376 | 457 | 441 |
| Scarlet Fever - - - - - - - - | 172 | 242 | 439 | 400 | 433 | 391 | 165 | 163 |
| Small Pox - - - - - - - - - | 100 | 152 | 40 | 216 | 426 | 52 | 40 | 281 |

It will be noticed that the diseases named in the above table ranged higher in 1854 than in 1853, with the exception of Scarlet Fever and Small Pox. In 1855 there has been a considerable decline in all of them except Small Pox, which has increased 70 per cent. above the number for 1854, or from 40 to 281.

With Croup there has been a steady increase from 1849 up to 1854. But during the last year, (1855) there has been a falling off of 6 per cent. Dysentery has fallen off since 1854, 25.28 per cent., Inflammation of the Brain 11.38 per cent., and Cholera Infantum 11.63 per cent.

Numerically, the deaths from Consumption rate fewer by 2.46 per cent. than those in 1854. But compared with the mortality for the year, they are 1 per cent. more. According to population they were 1 in every 376.03.

The number of deaths in each month of the year, according to sex, was as follows;

| Month. | Male. | Female. | Total. |
|---|---|---|---|
| January, | 450 | 416 | 866 |
| February, | 387 | 381 | 768 |
| March, | 481 | 411 | 892 |
| April, | 398 | 372 | 770 |
| May, | 491 | 418 | 909 |
| June, | 379 | 316 | 695 |
| July, | 596 | 517 | 1113 |
| August, | 779 | 676 | 1455 |
| September, | 441 | 378 | 819 |
| October, | 467 | 400 | 867 |
| November, | 311 | 276 | 587 |
| December, | 370 | 347 | 717 |
| | 5550 | 4908 | 10458 |

This table shows an excess of 642, or 6.14 per cent. of deaths in the males over the females. In 1854, the excess was 7.77 per cent.

In the total of deaths for the year will be found 1481 reported from the Alms House, from the colored population and from the Country, in the following proportions, according to the months:

| | Jan. | Feb. | March. | April. | May | June. | July. | Aug. | Sept. | Oct. | Nov. | Dec. |
|---|---|---|---|---|---|---|---|---|---|---|---|---|
| Alms House, | 72 | 86 | 76 | 65 | 73 | 52 | 65 | 92 | 53 | 40 | 37 | 48 |
| Blacks, | 45 | 36 | 59 | 70 | 61 | 41 | 56 | 81 | 37 | 50 | 26 | 26 |
| Country, | 5 | 6 | 8 | 5 | 8 | 11 | 16 | 24 | 27 | 16 | 5 | 3 |

Grand total—Alms House, 759 : Blacks, 588 : Country, 134.

From this table it will be seen, that the Alms House furnishes 7.15 per cent. of the mortality. The highest mortality in that institution for any one month was in August, 92; the lowest was in Nov., 37.

The colored population contributed 588 of the deaths, or 5.62 per cent.

The deaths from the Country amounted to 134, which number should properly be deducted from the total, as they do not belong to the city mortality.

